# *GNB3*, *eNOS*, and Mitochondrial DNA Polymorphisms Correlate to Natural Longevity in a Xinjiang Uygur Population

**DOI:** 10.1371/journal.pone.0081806

**Published:** 2013-12-20

**Authors:** Muyesai Nijiati, Abulajiang Saidaming, Jun Qiao, Zuheng Cheng, Changchun Qiu, Yujing Sun

**Affiliations:** 1 Cardiology Department of Internal Medicine, Xinjiang Uiygur Autonomous Region People's Hospital, Urumqi, China; 2 Department of Cardiac Surgery, First Affiliated Hospital, Xinjiang Medical University, Urumqi, China; 3 Institute of Basic Medical Science, CAMS & PUMC, Beijing, China; University Paris South, France

## Abstract

**Background:**

In centenarian populations, application of the positive biology approach (examination of positive phenotypes in aging) has revealed that mitochondrial DNA (mtDNA) mutation accumulation may be linked to human longevity; however, the role of guanine nucleotide-binding protein (G protein) abnormalities modulated by G-protein beta-3 (*GNB3*) and nitrate (NO2) production associated with endothelial nitric oxide synthase (*eNOS*), commonly appearing in age-related diseases, remains undetermined.

**Objective:**

The association between the mtDNA 5178A/C, mtDNA 10398A/G, *GNB3 C825T*, and *eNOS* polymorphisms and longevity in a Uygur population (Xinjiang region, China) were investigated.

**Methods:**

A total of 275 experimental subjects aged ≥100 or with 4 generations currently living were screened for inclusion in the centenarian (>100 years) and nonagenarian groups (90–100 years), and 112 65–70 year old control subjects were selected. Polymerase chain reaction-restriction fragment length polymorphism (PCR-RFLP) was used to examine mtDNA 5178A/C, mtDNA 10398A/G, *GNB3 C825T*, and *eNOS*. Associations between polymorphic loci, genotypes, and longevity were analyzed.

**Results:**

165 included subjects (M∶F = 107∶58; mean age = 97±3 years; mean age 100–113 years) were assigned to the centenarian (M∶F = 46/19; *n* = 65) and nonagenarian groups (M∶F = 61/39; *n* = 100). Associations between mtDNA C5178A and A10398G polymorphisms with longevity in the centenarian group with mtDNA genotype frequencies 5178A and 10398G were 66.79% and 36.8%.

**Conclusions:**

Applying the overwhelming longevity observed in Uygur populations, these findings demonstrate that mtDNA 5178A/C and 10398A/G, *GNB3 C825T*, and *eNOS* polymorphisms are useful as a genetic basis for longevity.

## Introduction

Human aging and longevity are of great interest to healthcare professionals and public policy makers worldwide. In China, there are nearly 48,000 citizens older than 100 years of age, exhibiting a recent growth rate of more than 10 percent according to Chinese Society of Gerontology [1]. Higher concentrations of older citizens tend to be found in the most populated countries, and more than 19.4 million of the oldest-old (>80 years of age) have been reported in China compared to only 12 million in the United States and 8.7 million in India [2]. In Singapore and Taiwan, more than 81% of citizens over 60 years of age have been reported to be of Chinese heritage [3]. Examining longevity and aging in heterogeneous Chinese populations may contribute to improving general understanding of aging mechanisms, and the design of future therapeutics to promote longevity in other populations.

Oldest-old age is variably defined as age in years >80 (octogenarians), >90 (nonagenarians), and >100 (centenarians) in studies of longevity, although these studies are often scare due to lack of sufficient cohorts of adults at advanced ages in certain populations [4]. It has been estimated that the number of centenarians worldwide will double each decade until 2100 [5], making these studies increasingly feasible and important in the future. However, analyses are often complicated by accurate age assessment. In fact, a recent review indicated that up to 90% of reported centenarians may have exaggerated their age, and age verification remains challenging in many regions due to poor, inconsistent, or missing public records [6].

While mortality increases with age through most of human adult life, in the oldest-old members of the population, mortality rates fail to exhibit a close and direct association with mortality [7]. This phenomenon may be due to the unique presence or absence of specific polymorphisms during aging, altering the rate of time-dependent cellular and system functionality deterioration [8]. As individuals drop out of the population, the distribution of a range of genotypes and other survival-related attributes dramatically changes with increasing age [9]. Thus, identification and characterization of these genotypes is critical to potentially delaying aging and increasing longevity.

The mitochondrial theory of aging suggests that a progressive buildup of somatic mutations in the mitochondria, the major site of cellular energy production, leads to a decline in mitochondrial function due to accumulating deletions and mutations in mitochondrial DNA (mtDNA) [10], [11]. Contemporary molecular biology techniques have been applied to screen longevity-associated susceptible genes. Polymorphisms of the displacement loop (D-loop) region of mtDNA and several serum parameters have been previously used to evaluate mtDNA in Chinese centenarians, indicating that mt146T is prevalent in the oldest-old and may be associated with lower systolic blood pressure, total cholesterol, triglyceride, and low density lipoprotein levels [4].

Other mtDNAs have also been explored. The mtDNA 5178 adenine/cytosine (mt5178 A/C) polymorphism (also known as NADH dehydrogenase subunit 2237 leucine/methionine [ND2-237 Leu/Met] polymorphism) has been investigated in association with longevity in a Japanese population [12]. The mt5178A genotype is a marker of mitochondrial haplogroup D that may have anti-atherogenic effects and reduce risk for adult-onset conditions, such as diabetes. In the Japanese population, the ratio of Mt5178 A/C was significantly lower in old patients than in both centenarians and healthy controls, suggesting that individuals with Mt5178C are more susceptible to adult-onset diseases compared to those with Mt5178A. [12]. Similar observations have not been reported in Chinese [4] or European [13] populations [4]. In fact, a comparison of Finnish and Japanese populations revealed the only common polymorphisms between populations were mt489C, mt10398G, and mt150T [13]. The mt10398 adenosine/guanine (A/G) polymorphism present in European haplogroups J, K, and Z has been associated with increased risk of invasive breast cancer in women of African descent, which may be linked to reduced longevity [14]. Notably, extensive controversy exists on the prevalence of mt5178C/A and mt10398A/G polymorphisms between populations of different nationalities and ages, with some studies reporting strong links with longevity and some reporting no link at all for various mitochondrial haplogroups [15].

Guanine nucleotide-binding proteins (G proteins) are a family of proteins that are extensively involved with receptor-coupled signal transduction systems, generally consisting of α, β, and γ subunits [16]. Guanine nucleotide binding protein beta-3 (*GNB3*) variants *C825T* and *Ser275Ser(M/N)* [rs5443] may be associated with hypertension elevation responsible for decreased longevity, and *GNB3* may be implicated in obesity, coronary artery disease, left ventricular hypertrophy, and diabetes mellitus [17]. *GNB3 C825T* and *ACE I/D* polymorphisms have also been linked with hypertension and other vascular dysfunctions in aging Korean patients [18]. G protein dysfunctions, potentially with a genetic basis have been associated with conditions that generally occur in older patients, such as Alzheimer's disease [19]. Furthermore, genetic regulation of G proteins affects receptor coupling to ionic channels and other effector systems in the prefrontal cortical membranes that may be related to sustained cognitive function and progressive cognitive dysfunction in older patients [19], [20]. Despite these potential links between G proteins and aging, few studies have explored the associations between *GNB3* variants and longevity.

Human longevity may also be impacted by nitric oxide synthases (NOSs), an enzyme family that catalyzes nitric oxide (NO) production from L-arginine that is critical to intracellular signaling [21]. Endothelial dysfunction caused by vascular damage is a hallmark sign of advanced age that may be modulated by NO, along with smooth muscle relaxation, inhibition of platelet aggregation, preservation of endothelial progenitor cell function, and leukocyte adhesion to endothelial cells [21], [22]. Insulin-like-growth-factor-1/insulin signal (IIS), primarily modulated by caloric intake and exercise, is a central regulator of endothelial nitric oxide synthase (*eNOS*) activation responsible for endogenous free-radical cellular stress due to production of excessive nitrates (NO_2_) [23]. Thus, *eNOS* may be critically involved in longevity by increasing the deteriorative effects of aging.

Examination of oldest-old, particularly centenarian, populations to determine the traits associated with longevity is referred to as the positive biology approach [24]. Using this approach, the current study investigates the associations between polymorphisms in mtDNA, *GNB3* (*C825T*), and *eNOS* in a Uygur population in the Hetian region of Xinjiang, China. Analysis of the genetic characteristics of these subjects in the Uygur population may contribute to the general understanding of genetic parameters associated with aging and longevity.

## Materials and Methods

### 2.1 Subjects

A total of 300 permanent residents in Hetian region (Xinjiang, China) were screened for inclusion in multiple epidemiological surveys conducted from October 1st 1999 through August 31st 2007 by examining life insurance data for subjects aged ≥100 or families with ≥4 living generations. 275 residents matched the selection criteria and were included in this study. Each subject was approached in-person by home visit, and participation was requested. Included subjects were assigned to the centenarian group (ages >100) or nonagenarian group (ages 90–100). A total of 112 subjects aged 65–70 years that died of apparently natural causes and reported no blood relations surviving to ages >70 years in the most recent two generations were used as a control group. The study protocol was approved by the Ethics Committee of the First Affiliated Hospital, Xinjiang Medical University, Urumqi, China. All included subjects provided signed informed consent for participation.

Inclusion of subjects in the experimental groups was determined using age validation criteria. Accordingly, control subjects were randomly selected and matched by gender and residence region.

### 2.2 Age validation

Subject age was validated using the five standards for age validation of centenarian and longevity, as previously described [6]. Briefly, age determination involved use of (1) the roster of centenarians provided by the life-insurance company; (2) verification of four generations through interviewing village and family members individually; and (3) using household age register data in 1981 (reported age) and subject self-assessment of age (presumptive age) during four major historical events, including the First Pandemic Plague in China (1902), Second Pandemic Plague in China (1912–1913), Anti-Japanese War by the Muslims ethnics (1936), and Chinese Land Reform and Collectivization (1952), and two personal events (marriage and military service). The error between reported age and presumptive age was assumed to be ≤5 years. For assessment of presumptive age by historical events, it was assumed that ages have the following characteristics: <6 years = no memory of events; 6–12 years = vague or incomplete memory of events; 12 years = complete memory of events. For assessment of presumptive age by personal events, it was assumed that marriage occurred at age ≥15 years, and military service entrance occurred at ages ≥18 years. Additionally, (4) age validation was verified by triplicate physical assessments by three independent physicians to verify presumptive age, with no contradictory reports between physicians. As a final step, (5) experimental ages used in the present study (actual ages) were calculated using the following formula:
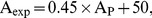
(1)where A_exp_ is the actual age used in this study, A_P_ is the self-reported presumptive age following physician verification. The error between actual age and self-reported age was assumed to be ≤3 years.

### 2.3 Sample collection

Initial whole blood samples were collected from each subject between October and November 1999. DNA samples were extracted and amplified from the blood provided by each subject. DNA samples were stored at −70°C.

Briefly, genomic DNA was isolated from 5 ml of peripheral venous blood collected after a 12 h fast and anticoagulated with 2% EDTA. Red blood cells were lysed using a hypotonic method, and white blood cells were isolated. Genomic DNA was extracted using conventional phenol/chloroform methods and an *A*_260_/*A*_280_ ratio ≥1.8.

### 2.4 Determination of mtDNA 5178A/C polymorphism

Primers for amplification of the mtDNA 5178A/C gene are shown in Table S1. Polymerase chain reaction-restriction fragment length polymorphism (PCR-RFLP) was performed in a 25-µl total volume containing 2.5 µl of 10× PCR buffer, 1 µl of 2 mmol/L dNTP, 2 µl of each primer, 0.5 µl of DMSO, 2 µl of template DNA, 0.25 µl of Taq polymerase (Shanghai Forward Biotech Co., Ltd., Shanghai, China), and 15.75 µl of ddH_2_O, using a programmable thermal controller (PTC-100; BioRad, MJ, USA) under the following conditions: an initial pre-denaturation at 94°C for 5 min, followed by 35 cycles of denaturation at 94°C for 45 s, annealing at 66°C for 45 s, and extension at 72°C for 45 s, with a final extension at 72°C for 5 min. The amplification fragment was 358 bp in length. About 10 µl of the PCR amplification product was digested with 0.25 µl of the restriction enzyme Alu I (Promega Corporation, Madison, WI, USA) in a water bath at 37°C for 3 h, and 10 µl of the digestive product and 2 µl of loading buffer were electrophoresed on a 3% agarose gel containing 0.5 mg/L ethidium bromide at 120 V for 15 min. The genotype was identified under an ultraviolet lamp. If the genotype CC occurred, two fragments with sizes of 307 bp and 51 bp were produced after digestion. If the genotype AA occurred, there was no restriction enzyme site, and only a fragment 358 bp in length was produced.

### 2.5 Determination of mtDNA 10398A/G polymorphism

Primers for amplification of the mtDNA 10398A/G gene are shown in Table S1. PCR-RFLP was performed in a 25-µl total volume containing 2.5 µl of 10× PCR buffer, 1 µl of 2 mmol/L dNTP, 2 µl of each primer, 0.5 µl of DMSO, 2 µl of template DNA, 0.25 µl of Taq polymerase (Shanghai Forward Biotech Co., Ltd., Shanghai, China), and 15.75 µl of ddH_2_O, using a programmable thermal controller (PTC-100; BioRad, MJ, USA) under the following conditions: an initial pre-denaturation at 94°C for 5 min, followed by 30 cycles of denaturation at 94°C for 30 s, annealing at 52°C for 30 s, and extension at 72°C for 30 s, with a final extension at 72°C for 5 min. The amplification fragment was 101 bp in length. About 10 µl of the PCR amplification product was digested with 0.25 µl of the restriction enzyme Bgl I (Promega Corporation, Madison, WI, USA) in a water bath at 37°C for 5 h, and 10 µl of the digestive product and 2 µl of loading buffer were electrophoresed on a 10% polyacrylamide gel at 160 V for 5 h. The electrophoresis product was analyzed by silver nitrate staining, and the genotype was identified. If the genotype GG occurred, two fragments with sizes 83 bp and 16 bp were produced after digestion. If the genotype AA occurred, only a fragment 101 bp in length was produced.

### 2.6 Determination of *GNB3 C825T* polymorphism

PCR-RFLP was employed to detect the genotype of *GNB3 C825T* polymorphism using the primers shown in Table S1. PCR-RFLP was performed in a 25-µl total volume containing 0.01–0.05 µg of template DNA, 2 µl of each primer, 1 µl of 2 mmol/L dNTP, 0.5 U of Taq polymerase (Shanghai Forward Biotech Co., Ltd., Shanghai, China), 2.5 µl of 10× Tris-HCl buffer, and sterile ddH_2_O, using a programmable thermal controller (PTC-100; BioRad, MJ, USA) under the following conditions: an initial pre-denaturation at 94°C for 5 min, followed by 35 cycles of denaturation at 94°C for 60 s, annealing at 66°C for 45 s, and extension at 72°C for 60 s, with a final extension at 72°C for 7 min. The PCR amplification product was digested with the restriction enzyme BseD I (Promega Corporation, Madison, WI, USA) in a water bath at 55°C for 3 h, and electrophoresed on a 2% agarose gel. The genotype was identified under an ultraviolet lamp. Two bands of sizes 152 and 116 bp indicated the genotype CC, three bands with sizes of 268, 152, and 116 bp indicated the genotype CT, and only one band sized 268 bp indicated the genotype TT. In addition, 50 µl of the PCR amplification product was collected and sequenced by the BGI LifeTech Co., Ltd. (Beijing, China).

### 2.7 Determination of biochemical parameters

Biochemical parameters of each blood sample were determined using a fully automated biochemistry analyzer, including total cholesterol (TC), triglycerides (TG), high-density-lipoprotein (HDL) cholesterol, low-density-lipoprotein (LDL) cholesterol, apolipoprotein A (Apo A), apolipoprotein B (Apo B), and blood glucose. Physical examinations included measurement of blood pressure (seated blood pressure), determination of blood biochemical parameters, electrocardiography, cardiopulmonary examination, and smoking and alcohol status. Medical history, physical examinations, and laboratorial examinations, including measurement of height, body weight, and systolic and diastolic blood pressure were recorded for all patients.

### 2.8 Determination of eNOS polymorphism and alleles

Phenol/chloroform was used to extract genomic DNA from peripheral blood leucocytes that had been lysed with sodium dodecyl sulfate and treated with proteinase K. PCR amplifications were performed in a PTC-200TM therameter. The primers for PCR amplifications were designed based on the published sequence of eNOS (http://www.ncbi.nlm.nih.gov/Genbank) accession number X76307.1.

Genetic variants containing the T-786C polymorphism were determined by PCR amplification using specific primers (Table S2).These PCR products were digested with Msp I for 3 h at 37°C. The fragments were separated by electrophoresis in a 10% polyacrylamide gel and were visualized by silver staining. The G298T polymorphism in exon 7 was detected using specific primers (Table S2). A 457 bp PCR fragment containing this polymorphism was digested with Mbo I for 3 h at 37°C. Products were electrophoresed on a 3% agarose gel and visualized by ethidium bromide staining. Genotypes containing the 27 bp-repeat in intron 4 of the eNOS gene were identified by PCR using specific primers (Table S2). PCR fragments were separated by electrophoresis in a 10% polyacrylamide gel and visualized by silver staining.

### 2.9 Statistical analysis

Gene frequency was calculated by gene counting, and all statistical analyses were performed using SAS version 9.1.3 (SAS Institute Inc., Cary, NC, USA). Hardy-Weinberg equilibrium was tested using chi-square tests and Finetti method. Associations between polymorphic loci and longevity were estimated using the chi-square test and Fisher's exact test. The association between genotypes and longevity was evaluated using analysis of covariance (ANCOVA) and stepwise logistic regression analysis, and the probability, odds ratio (*OR*), and 95% confidence interval (*CI*). *P*-values less than 0.05 were considered statistically significant (*P*<0.05).

## Results

### 3.1 Clinical and demographic characteristics of included subjects

275 of the 300 patients screened agreed to provide consent for participation in the study, and 25 were excluded for failure to consent to participation. Of the 275 subjects, 165 met all criteria for inclusion (M∶F = 107∶58; mean age = 97±3 years; age range 100–113 years). These patients were further divided into the centenarian (M∶F = 46/19; *n* = 65) and nonagenarian groups (M∶F = 61/39; *n* = 65). There were a total of 112 control group subjects (M∶F = 53∶59; mean age = 67±3 years; age range = 65–70 years). Notably, no significant associations between alleles and blood lipid levels were observed in any group (*P*>0.05). Other than age (P<0.05), no significant differences were observed in clinical or demographic parameters between groups (all *P*>0.05) (Table 1).

**Table 1:** Clinical and demographic parameters (mean ± SD).

*Clinical characteristic*	*Centenarian group (n = 65)*	*Nonagenarian group (n = 100)*	*Control group (n = 112)*	*P*-value
Age (years)	100–113	91–99	67±3	<0.05¶ ‡
Gender (male/female)	46/19	61/39	53/59	>0.05
Body mass index(kg/m^2^)	19.64±2.77	20.21±2.25	21.29±2.49	>0.05
Systolic blood pressure (mmHg)	115.26±8.93	115.64±8.65	116.82±8.83	>0.05
Diastolic blood pressure (mmHg)	68.62±5.37	69.56±5.22	70.62±5.41	>0.05
TC (mmol/L)	4.80±1.09	4.82±1.03	4.76±0.97	>0.05
HDL cholesterol (mmol/L)	1.03±0.26	1.10±0.27	1.10±0.28	>0.05
LDL cholesterol (mmol/L)	3.38±1.09	3.05±0.82	2.95±0.98	>0.05
Apo A (g/L)	1.29±0.22	1.26±0.19	1.32±0.19	>0.05
Apo B (g/L)	1.01±0.31	0.98±0.24	1.15±0.54	>0.05
TG (mmol/L)	1.65±0.75	1.67±0.73	1.81±0.78	>0.05
Fasting blood glucose (mmol/L)	5.91±2.18	5.93±2.14	5.71±1.19	>0.05

*Note*: Levels of all clinical biochemical parameters were expressed by mean ± SD. *P*-value indicates the difference between the three groups determined by ANOVA.

¶ Control vs. nonagenarian group;

‡ Control vs. centenarian group.

### 3.2 Frequency of polymorphic mtDNA loci 5178A/C and *10398G/A*

Determination of the mtDNA 5178A/C polymorphism using PCR-RFLP showed that the length of the amplification fragment was 358 bp, producing the two genotypes AA and CC after enzyme digestion. The 10398 locus in mtDNA had a total length of 101 bp, producing the two genotypes AA and GG after enzyme digestion. The frequencies of mtDNA variations 5178A and 10398G in the centenarian group were 66.79% and 36.8%, respectively.

There were no significant differences in the frequency of the genotypes of the polymorphic loci 5178A/C and 10398G/A between the centenarian group and the nonagenarian group, while significant differences were detected between the centenarian, nonagenarian groups, and the control group (for the locus mt5178A/C, χ^2^ = 8.8515, *P* = 0.0029 centenarian group vs. control group, χ^2^ = 4.9424, *P* = 0.0262 nonagenarian group vs. control group; for the locus 10398G/A, χ^2^ = 6.0393, *P* = 0.0140 centenarian group vs. control group, χ^2^ = 4.5974, *P* = 0.0032 nonagenarian group vs. control group). Examination of the genotype frequency of polymorphic loci in mtDNA revealed a significant difference for A/C as well as A/G in genotype C5178A between the centenarian and nonagenarian groups (*P* = 0.0029 and P = 0.0140, respectively). A significant difference was also observed for A/C in genotype C5178A (*P* = 0.0262) and A/C in genotype A10398G (*P* = 0.0320) between the nonagenarian and control groups (Table 2).

**Table 2:** Genotype frequency of polymorphic mtDNA loci C5178A and A10398G.

*Group*	*Genotype C5178A*				Genotype A10398G			
	A	C	?^2^ value	*P*-value	A	G	?^2^ value	*P*-value
Centenarian (*n* = 65)	51	14	1.14	0.2860†	18	47	0.35	0.5563†
Nonagenarian (*n* = 100)	71	29	8.85	0.0029‡	32	68	6.04	0.0140‡
Control (n = 112)	63	49	4.94	0.0262¶	52	60	4.60	0.0320¶

*Note*: Comparison of genotype or allele.

† Centenarian vs. nonagenarian group;

‡ Centenarian vs. control group;

¶ Nonagenarian vs. control group.

Linkage disequilibrium analysis revealed four haplotypes in each group, and there were no significant differences in the frequencies of the four haplotypes between the centenarian group and the nonagenarian group. However, the haplotype frequencies of mtDNA 5178C/A and 10398A/G in the centenarian group and the nonagenarian group were significantly higher than those in the control group (Table 3).

**Table 3:** Frequency of haplotypes of polymorphic loci in mtDNA.

*Haplotype*		*Frequency by group*			*P-value* ‡	*P*-value¶
5178A/C	10398G/A	Centenarian	Nonagenarian	Control		
A	G	38	50	41	0.0048	0.1615
C	G	9	18	19	0.5837	0.8428
C	A	5	11	30	0.0021	0.0037
A	A	13	21	22	0.9541	0.8062

‡ Centenarian vs. control group;

¶ Nonagenarian vs. control group.

The *C825T* mtDNA polymorphism was localized within exon 10 of the *GNB3* gene by comparing the DNA from peripheral blood cells of hypertensive patients and controls.

### 3.3 Frequency of genotype of *GNB3 825C/T* polymorphism and allele

The distribution of the *GNB3* genotypes in the centenarian group and the nonagenarian group conformed to Hardy-Weinberg equilibrium (*P* = 0.4668 and 0.1592), while the frequency of the *GNB3* genotype in the control group did not conform to Hardy-Weinberg equilibrium (*P* = 0.0265). There were significant differences in the frequencies of the genotypes and alleles of *GNB3 825C/T* between the centenarian group and the control group (*P*<0.01), and the most significant differences were detected in the frequency of the genotype CC (*OR* = 2.60, 95% *CI* = 1.39–4.89). In addition, significant differences were observed in the frequencies of the genotypes and alleles of *GNB3 825C/T* between the nonagenarian and control group (*P*<0.01 and *P*<0.05, respectively), and the frequency of the genotype CC was 51.0% in the nonagenarian group (*OR* = 1.80, 95% *CI* = 1.04–3.12). However, there were no significant differences found in the frequencies of the genotypes and alleles of *GNB3 825C/T* between the centenarian group and the nonagenarian group (Table 4).

**Table 4:** Genotype frequency of polymorphic locus *825C/T* in the *GNB3* gene.

*Group*	*Genotype*									Allele	
	CC			CT			TT			C	T
	Frequency	OR	CI	Frequency	OR	CI	Frequency	OR	CI		
Control	41 (36.6)	2.60‡	1.39–4.89‡	36 (32.1)	0.87‡	0.45–1.7‡	35 (31.3)	0.27‡	0.11–0.6‡	118 (52.7)	106 (47.3)
Centenarian	39 (60.0)**			19 (29.2)**			7 (10.8)**			97 (74.6)**	33 (25.4)**
Nonagenarian	51 (51.0)*	1.80¶	1.04–3.12¶	32 (32.0)*	0.99¶	0.56–1.77¶	17 (17.0)*	0.45¶	0.23–0.87¶	134 (67.0)**	66 (33.0)**

*Note*: 95% CI was used.

* *P*<0.05 vs. control group;

** *P*<0.01 vs. control group.

‡ Centenarian vs. control group;

¶ Nonagenarian vs. control group.

### 3.4 Frequency of genotypes of *eNOS* polymorphism and alleles

For *eNOS* 298, significant genotypic differences but not allele frequencies were observed between the centenarian group and control group, with TT higher in the control group. No significant differences were observed between the nonagenarian group and the control group. No significant differences were observed between *eNOS* 786 and 27 in genotype or allele frequencies among all three groups (Table 5).

**Table 5:** Genotype frequency of polymorphic *eNOS*.

*Group*	*eNOS 298 genotype*				
	GG	GT	TT	G	T
Centenarian	23 (35.4%)**	42 (64.6%)**	0(0)**	88 (67.7%)	42 (32.3%)
Nonagenarian	48 (48.0%)	45 (45.0%)	7 (7.0%)	141 (70.5%)	59 (29.5%)
Control	57 (50.9%)	52 (46.4%)	3 (2.7%)	166 (74.1%)	58 (25.8%)

	*eNOS -786* genotype				
	TT	TC	CC	T	C
Centenarian	43 (20.6%)	81 (38.8%)	85 (40.7%)	165 (39.7%)	251 (60.3%)
Nonagenarian	22 (33.3%)	19 (28.8%)	25 (37.9%) 2(100.0%)	63 (47.7%) 0(0)	69 (52.3%) 4(100%)
Control	0 (0)	0 (0)			

	*eNOS 27* genotype				
	II	ID	DD	I	D
Centenarian	49 (22.4%)	78 (35.6%)	92 (42.0%)	176 (40.2%)	262 (59.8%)
Nonagenarian	14 (26.4%)	20 (37.7%)	19 (35.8%)	48 (45.3%)	58 (54.7%)
Control	2 (40.0%)	2 (40.0%)	1 (20.0%)	6 (60.0%)	4 (40.0%)

** *P*<0.05 vs. control group.

## Discussion

The current study determined the genotypes of *GNB3 T825C* in Uygur centenarians, nonagenarians, and control subjects in the Hetian region (Xinjiang, China) and evaluated the associations between *GNB3 T825C* polymorphism and longevity. Associations between mtDNA C5178A and A10398G polymorphisms with longevity were observed in Uygur centenarians with genotype frequencies for 5178A and 10398G of 66.79% and 36.8%, respectively. Additionally, relationships between *GNB3* and *eNOS* and longevity were ascertained, indicating that these genes may be involved in achieving oldest-old ages of >90 and >100 years in Chinese populations. Due to the overwhelming longevity observed in Uygur populations [25], this data provides a basis for further comparison between other populations and age groups that may indicate important genetic contributors for longevity and deteriorative aging processes, potentially providing useful targets for further research and therapeutic development. Uygur populations evidence genetic phenotypes associated with lower rates of chronic diseases, cancer, and age-related diseases, such as Alzheimer's and hypertension. Furthermore, eNOS 298 without TT may be correlated to longer life without dementia, meriting further study with the potential to reveal genotypes associated with longer life.

The Uygur residents of the Taklimakan Desert are relatively isolated from outside influences, and these populations are primarily agrarian with only moderate daily nutrition consisting of little fat and abundant fruit sugars [25]. One of the most obvious issues in assessment of remote populations is that age is difficult to validate [8], as the modern Uygur registry for official age data was only adopted in 1949 [25]. More men than women centenarians have been reported in Uygur populations [25], consistent with the current findings. A study conducted by the Statistical Bureau of the Xiangjiang Uygur Autonomous Province (1985) also found that the vast majority of Chinese centenarians were of Uygur nationality residing in the Hetian region (802/865), while centenarians were much less common in Tajik (18/865), Kirghiz (14/865), Kazakh (12/865), Han (7/865), and Mongol (1/865) nationalities [25]. Thus, a strong genetic component to longevity was suspected and confirmed by the current findings in this particular Chinese population that may be of wide interest, pending further comprehensive evaluation.

A recent review compiled a comprehensive listing of genes reported to be associated with exceptional longevity (EL) in modern scientific literature, including GNB3 and the α-subunit of the Gs protein (GNAS1) [16]. These findings are consistent with the current results, though the associations between *eNOS* variants and longevity examined in the current study have not been previously documented. In addition, a Bama County (Guangxi, China), a region known for longevity, underwent full sequence analysis of mitochondrial DNA (mtDNA), associating mitochondrial variations A73G, A263G, A2076G, A8860G, G11719A, C14766T, and A15326G as well as haplogroups M^*^, F1, D*, and D_4_ with longevity [4]. Though scattered data is currently available in the literature regarding several of these genes, improved documentation of the genes proposed in these studies in diverse populations may offer useful targets for prevention, treatment development, and risk assessment for chronic diseases that commonly appear later in life, due to the fact that oldest-old patients generally exhibit lower rates of cardiovascular disease, diabetes mellitus, Alzheimer's, and cancer [19].

*GNB3*, encoding 340 amino acids, is located on chromosome 12p13 and consists of 11 exons, with a relative molecular mass of 37×10^3^ [16], [17]. The allele T causes loss of 498–620 bases on the mRNA sequence transcribed by the *GNB3* gene, thereby resulting in the production of a splicing variant of *GNB3*, Gß3-s [26], [27]. Compared with the wild-type *GNB3* gene, this variant lacks 41 amino acids and 1 WD repeat domain. After Gß3-s binds to the Gα1 and Gβ subunits, excessive G protein activation occurs that may promote vasoactive substances and growth factors, resulting in intense vasoconstriction and proliferation of vascular smooth muscle cells [27]. The human *GNB3 C825T* polymorphism accelerates intracellular information transfer, enhances the activity of intracellular sodium/hydrogen exchange, and increases the risk of hypertension and individual blood pressure-related organ damage [17]. However, inconsistent results have been obtained from the studies of *GNB3 C825T* polymorphisms conducted in different ethnic populations, with the highest *GNB3 C825T* allele frequencies reported in black African populations, who are also especially prone to obesity and hypertension in Western societies [14]. Consistent with these reports, the current study revealed that higher *GNB3 C825T* allele frequencies in the control group corresponded with shorter life. Chronic and age-related diseases, such as obesity and hypertension, may also reduce life expectation. Centenarians and long-lived individuals with *GNB3 T825C* genotype were less likely to exhibit these chronic diseases, which may have contributed to longer life expectation.

It has been demonstrated that mtDNA 5178A/C and *10398G/A* polymorphisms may affect the aging process [12]; however, the underlying mechanism of this association remains unclear. MtDNA divergence occurs 20-fold more rapidly than that observed in nuclear DNA due to high fluorine pressure, lipophilic cation aggravation, and extremely frequent attacks by fluorine radicals associated with the respiratory chain [28]. Because mitochondria are the main source of reactive oxygen species (ROS) in mammalian cells, oxidative damage may be implicated is altering cell lifecycles, increasing the rate of sequence substitutions in the mtDNA control region (CR), and causing energy deficiency through yet poorly characterized indirect mechanisms [28]. Mitochondria that undergo oxidative stress, calcium overload, and ATP depletion have recently been demonstrated to undergo a “a permeability transition,” wherein the inner mitochondrial membrane permeability for water and low-molecular weight solutes (≤1.5 kDa) rapidly changes causing oxidative phosphorylation uncoupling as well as membrane depolarization [29]. Cumulatively, this process has been termed mitochondrial permeability transition pore (MPTP), and it may play a key role in cellular life, death, and aging processes [29]. The current study results suggest that different genotypes in mtDNA may have different effects on MPTP, thus affecting aging processes. Although further experiments are required to support this mechanism, these results provide a basis for future studies.

Polymorphisms of the *eNOS* gene have been linked with chronic health conditions, including type II diabetes [30] and abnormal NO production leading to increased susceptibility to coronary artery disease [31]. Furthermore, Hingorani *et al*. [32] reported a significant association between *eNOS* variants and hypertension, with carriers of *eNOS Asp298* at elevated risk of developing pre-eclampsia and adaptive vascular changes during pregnancy [32]. In some patients with specific *eNOS* variants, in particular the *Glu298Asp* variant, mental stress produces a much more pronounced change in vascular flow, most noticeable in patients with other endothelial dysfunction [33]. Xin *et al*. [34] recently reported that homozygosity for +G894T (E298D) in *NOS3* increases left ventricular hypertrophy risk in hypertension patients. Aging patients may be potentially susceptible to *eNOS-*induced abnormal NO signaling in the brain, which has been linked a variety of neurodegenerative pathologies such as stroke and excitotoxicity, Alzheimer's disease, multiple sclerosis, and Parkinson's disease, conditions responsible for dementia in a large portion of the geriatric population [35]. Thus, variations between centenarians and the control group may be related to *eNOS* polymorphisms, making centenarian populations less susceptible to a variety of progressive, age-related conditions, thereby contributing to the longevity of these populations due to gene dosage effects. Conversely, other *eNOS* polymorphisms may increase the susceptibility to these progressive, age-related disorders, thereby reducing the longevity.

The current study results must, however, be considered as preliminary due to the subjective nature of the validation method, which cannot confirm true age with complete accuracy. Additionally, these findings may be confounded by environmental considered in the Hetian region, such as relatively low socioeconomic conditions, low population mobility, limited access to healthcare, limited diet, and high population homogeneity [25]. Discrepancies between male and female centenarian numbers do not consider the implications of limited access to modern healthcare in the Hetian region [25], which may account for higher levels of death due to environmental considerations and routine pregnancy complications in females that do not accurately correlate longevity with genetic characteristics. Further epidemiological and genetic studies will be required to assess the relationship between environmental and genetic factors in longevity and aging mechanisms. This study, does, however, indicate that the proposed genotypes are interesting targets for further longevity-related research, meriting examination in other, diverse patient populations.

To our knowledge, this is the first study to evaluate the association between *GNB3 825* polymorphisms and longevity in the Uygur population of China. Cumulatively, it indicates that associations exist between mtDNA 5178A/C and 10398G/A polymorphisms and longevity. Furthermore, eNOS 298 without TT may be correlated to longer life without dementia, and variations in the frequencies of genotypes and alleles of *GNB3 825C/T* between the centenarian group and the control group suggest a strong association between the genotype CC of the polymorphic locus 825CC in the *GNB3* gene and the longevity of Uygur persons. Further comprehensive, multidisciplinary, systematic studies will be required to investigate and confirm these associations and their relevant interactions in other populations.

## Supporting Information

Table S1 **PCR-RFLP amplification primers for each gene.**
(DOC)

Table S2 **Primers sequence and PCR amplification conditions for genotyping eNOS 3 polymorphisms.**
(DOC)
