# Supplementary material for: GNB3, eNOS, and Mitochondrial DNA Polymorphisms Correlate to Natural Longevity in a Xinjiang Uygur Population
Source: PLoS One. 2013 Dec 20;8(12):e81806. doi: 10.1371/journal.pone.0081806 (PMC3869651; doi:10.1371/journal.pone.0081806)
Supplement: Table S1 — PCR-RFLP amplification primers for each gene. (DOC) [file pone.0081806.s001.doc]

**Supplementary tables**

**Supplementary Table 1. PCR-RFLP amplification primers for each gene**

| *Gene* | *Primer* | *Sequence* |
| --- | --- | --- |
| **mtDNA 5178A/C** | forward | 5′-CTTAGCATACTCCTCAATTACCC-3′ |
|  | reverse | 5′-CTGAATTCTTCGATAATGGCCCA-3′ |
| **mtDNA 10398A/G** | forward | 5′-GTTTAAACTATATGCCAATTCGG-3′ |
|  | reverse | 5′- TTATGTCATCCCTCTTATTAA -3′ |
| ***GNB3 C825T*** | sense | 5′-TGA CCC ACT TGC CAC CCG TGC-3′ |
|  | anti-sense | 5′-GCA GCA GCC AGG GCT GGC-3′ |

*all primers were provided by BGI LifeTech Co., Ltd. (Beijing, China)
