# Supplementary material for: GNB3, eNOS, and Mitochondrial DNA Polymorphisms Correlate to Natural Longevity in a Xinjiang Uygur Population
Source: PLoS One. 2013 Dec 20;8(12):e81806. doi: 10.1371/journal.pone.0081806 (PMC3869651; doi:10.1371/journal.pone.0081806)
Supplement: Table S2 — Primers sequence and PCR amplification conditions for genotyping eNOS 3 polymorphisms. (DOC) [file pone.0081806.s002.doc]

**Supplementary Table 2. Primers sequence and PCR amplification conditions for genotyping eNOS 3 polymorphisms**.

| Polymorphism | Primers (5’-3’) | Tm (℃) | PAGE agarose | Restriction  enzyme | Fragments (bp) |
| --- | --- | --- | --- | --- | --- |
| T-786C | F: AGTTTCCCTAGTCCCCATGC  R:CACACCCCCATGACTCAAGT | 60℃ | 10% PAGE | PCR/RFLP  Msp I | T: 140+40  C:90+50+40 |
| Intron4  27bp repeat | F: GGCCCTATGGTAGTGCCTTG  R: TCTCTTAGTGCTGTGGTCAC | 55℃ | 10% PAGE |  | a 393; b 420;  c 447 |
| 298G/T | F:TCCCTGAGGAGGGCATGAGGC  R:TGAGGGTCGCACAGGTTCCT | 55℃ | 3%  Agarose | PCR/RFLP  Mbo I | G:457  T:320+137 |

PAGE indicates polyacrylamide gel electrophoresis.
